# Impact of SARS-CoV-2 vaccination on systemic immune responses in people living with HIV

**DOI:** 10.3389/fimmu.2022.1049070

**Published:** 2022-12-02

**Authors:** Clara Bessen, Carlos Plaza-Sirvent, Agit Simsek, Jaydeep Bhat, Corinna Marheinecke, Doris Urlaub, Petra Bonowitz, Sandra Busse, Sabrina Schumann, Elena Vidal Blanco, Adriane Skaletz-Rorowski, Norbert H. Brockmeyer, Oliver Overheu, Anke Reinacher-Schick, Simon Faissner, Carsten Watzl, Stephanie Pfaender, Anja Potthoff, Ingo Schmitz

**Affiliations:** ^1^ Department of Molecular Immunology, Ruhr University, Bochum, Germany; ^2^ Department of Molecular and Medical Virology, Ruhr-University Bochum, Bochum, Germany; ^3^ Department for Immunology, Leibniz Research Centre for Working Environment and Human Factors (IfADo) at TU Dortmund, Dortmund, Germany; ^4^ WIR - Walk In Ruhr, Center for Sexual Health and Medicine, Bochum, Germany; ^5^ Department of Dermatology, Venereology and Allergology, Interdisciplinary Immunological Outpatient Clinic, Center for Sexual Health and Medicine, Ruhr-Universität Bochum, Bochum, Germany; ^6^ Department of Hematology, Oncology with Palliative Care, St. Josef Hospital, Ruhr University Bochum, Bochum, Germany; ^7^ Department of Neurology, Ruhr-University Bochum, St. Josef Hospital, Bochum, Germany

**Keywords:** SARS-CoV-2, mRNA vaccine, HIV, immunodeficiency, antibody, T cells

## Abstract

Despite the development of vaccines, which protect healthy people from severe and life-threatening Covid-19, the immunological responses of people with secondary immunodeficiencies to these vaccines remain incompletely understood. Here, we investigated the humoral and cellular immune responses elicited by mRNA-based SARS-CoV-2 vaccines in a cohort of people living with HIV (PLWH) receiving anti-retroviral therapy. While antibody responses in PLWH increased progressively after each vaccination, they were significantly reduced compared to the HIV-negative control group. This was particularly noteworthy for the Delta and Omicron variants. In contrast, CD4+ Th cell responses exhibited a vaccination-dependent increase, which was comparable in both groups. Interestingly, CD4+ T cell activation negatively correlated with the CD4 to CD8 ratio, indicating that low CD4+ T cell numbers do not necessarily interfere with cellular immune responses. Our data demonstrate that despite the lower CD4+ T cell counts SARS-CoV-2 vaccination results in potent cellular immune responses in PLWH. However, the reduced humoral response also provides strong evidence to consider PLWH as vulnerable group and suggests subsequent vaccinations being required to enhance their protection against COVID-19.

## Introduction

Since early 2020, the world is suffering from a pandemic caused by severe acute respiratory syndrome coronavirus 2 (SARS-CoV-2, also known as 2019-nCoV) (1). Clinical manifestations of coronavirus disease 2019 (COVID-19), the disease caused by SARS-CoV-2, diverge from asymptomatic or mild influenza-like symptoms *via* hospitalization to death due to the acute respiratory failure. Most infected patients recover without the need for hospital care, but factors such as age or existence of comorbidities, like diabetes or immunodeficiency, determine the probability to develop severe COVID-19 (2, 3). SARS-CoV-2 infection elicits humoral responses, most importantly antibodies against viral proteins, as well as a wide variety of cellular responses (4–10). Among other measures to control the pandemic, vaccination strategies have been implemented to prevent severe COVID-19 (11). In contrast to traditional vaccination strategies, a novel technology of mRNA-based vaccines has been implemented. The novel mRNA-based vaccines deliver *in vitro* transcribed mRNA molecules encoding for a pathogen antigen, which are encapsulated in lipid nanoparticles (12). In case of COVID-19 mRNA-based vaccines, the SARS-CoV-2 spike protein, which binds to the human angiotensin-converting enzyme 2 (ACE2) receptor, is translated and presented by antigen-presenting cells, generating a specific immune response (12, 13). Besides safety and efficacy, studies demonstrated that these vaccines are able to generate a durable response (14–18). This immune response comprises the induction of germinal centers and production of vaccine-induced antibodies as well as the generation of spike-specific T cells (17, 19, 20). Nevertheless, due to a decline in the antibody titers over time and the rise of SARS-CoV-2 variants of concern, a vaccine booster dose is recommended to preserve the protective effect against COVID-19 (21–23).

Vaccine safety and efficacy are being stringently monitored during vaccine development and vaccination campaign. The immune response towards COVID-19 vaccines in patients with severe diseases such as cancer has recently been assessed (24–27). However, an in-depth analysis of immune responses in people living with HIV (PLWH) has not been performed, yet. Currently, it is poorly understood whether PLWH have a higher risk to develop severe COVID-19. In initial COVID-19 cohort studies, comparable clinical outcomes were found in the PLWH and HIV-negative individuals (28–34). Contrariwise, in other cohort studies, PLWH presented worse outcomes including higher rates of hospitalization and mortality (35–39). In addition, an observational study described lower IgG concentration and neutralizing antibody titers correlating with more cases of severe COVID-19 in PLWH (40). In contrast, a recent work investigating humoral and T cell specific-responses to the SARS-CoV-2 infection revealed that PLWH could mount a persistent immune response comparable to HIV-negative subjects (41). Additionally, the correlation analysis in the latter study suggested that deviations in the CD4 to CD8 ratio may result in a diminished ability to respond to SARS-CoV-2 infection in the PLWH (41). Taken together, the current data on SARS-CoV-2 infection and PLWH raises the question whether and how repeated COVID-19 vaccination raise immune responses to SARS-CoV-2 and especially towards current variants of concern (VOCs) such as Delta and Omicron. Our present study aims to clarify whether immunity elicited by a COVID-19 mRNA vaccine in the PLWH is modulated in comparison to vaccination in HIV-negative people.

## Material and methods

### Study design and ethics statement

Study participants were recruited at the WIR – Walk in Ruhr, Department of Dermatology, Ruhr-University Bochum. We included age-matched HIV-negative participants (controls; n=20) and PLWH (n=71). The written informed consent was obtained from all study participants. The clinical information of the study participants is presented in **Supplementary Table 1**. Samples from PLWH were collected immediately before the first vaccination (T0), at the day of second vaccination (T1) within 4-6 weeks (T2) following the second vaccination, at the day of third vaccination (T3) and within 4-6 weeks (T4) following the third vaccination against SARS-CoV-2. Similarly, samples from HIV-negative controls were collected at T2, T3 and T4. No control samples were available for the T0 and T1 time points.

### Cell isolation and cryopreservation

Blood collection for peripheral blood mononuclear cells (PBMC) isolation was conducted using KABEVETTE^®^ G EDTA tubes. 2.5 ml of blood was centrifuged at 1500 g for 10 min., the plasma was obtained and stored at -80°C until further use. The remaining blood was diluted 1:1 with PBS and slowly placed on top of Pancoll human (PAN-Biotech). Density gradient centrifugation was performed at 800 g for 30 min. without break. The interface containing the PBMCs was collected from the gradient and washed twice with PBS at 500 g for 10 minutes. The cell pellet was resuspended in PBS and the cell number was determined in a Sysmex KX-21N (Sysmex Europe GmbH). 1.8x10^7^ cells were cryopreserved in FCS (PAN-Biotech) containing 10% DMSO. The cryotubes were cooled down overnight in a Mr. Frosty (Sigma) at -80°C and stored in liquid nitrogen until further use.

### Anti-SARS-CoV-2 spike antibody titer

Plasma samples were analyzed for the spike (receptor-binding domain [RBD]; sequence derived from the original wild type SARS-CoV-2 strain) specific immunoglobulin G antibodies by enzyme-linked immunosorbent assay (ELISA) as described previously (42). Briefly, samples were diluted from 1:100 to 1:12,500 and results were expressed as the dilution, which still gave the same signal as an internal calibrator of the ELISA, indicating a positive result. The values obtained for samples below the detection limit were interpreted as negative and set to 1. The assay was calibrated according to the World Health Organization international standards and values were expressed as binding antibody units (BAU).

### SARS-CoV-2 neutralization assay

SARS-CoV-2 pseudoviruses were prepared as described previously (43). Briefly, sera were incubated for 30 min at 56°C in order to inactivate complement factors. Single cycle VSV∗ΔG(FLuc) pseudoviruses bearing the SARS-CoV-2 spike (D614G) protein (44), SARS-CoV-2 B.1.617.2 (Delta) (EPI_ISL_1921353) or SARS-CoV-2 B.1.1.529 (Omicron) (EPI_ISL_6640919) spike in the envelope were incubated with quadruplicates of two-fold serial dilutions from 1:20 to 1:2560 of immune sera in 96-well plates prior to infection of Vero E6 cells (1x10^4^ cells/well) in DMEM with 10% FBS (Life Technologies). 18 hours post infection, firefly luciferase (FLuc) reporter activity was determined as previously described (45) using a CentroXS LB960 (Berthold). The reciprocal antibody dilution causing 50% inhibition of the luciferase reporter was calculated as pseudovirus neutralization dose 50% (PVND50). Detection range is defined to be between 1:20 and 1:2560.

### PBMC stimulation using SARS-CoV-2 peptide pool

PBMCs were thawed in a 37°C water bath and diluted in 10 ml RPMI 1640 with Glutamine (Capricorn) with 5% human AB serum (PAN-Biotech) and 5 U/ml Benzonase (Merck/Sigma) and centrifuged at 400 g for 5 min. The pellet was washed with 10 ml thawing medium, resuspended in 5 ml culture medium (RPMI 1640 with Glutamine (Capricorn) plus 5% human AB serum (PAN-Biotech)) and cells were rested for at least 2 hours. Cell number was determined using 7-aminoactinomycin D in a Cytoflex LX (Beckman Coulter, RRID: SCR_019627). 100 µl per sample were seeded in duplicates in a flat bottom 96 well plate (Sarstedt) at a concentration of 1x10^7^ cells/ml. Cells were stimulated with PepTivator pools (Miltenyi Biotec) for 16 hours according to manufacturer’s instructions. The peptide mixes consisted of a pool of mainly 15-mer sequences with 11 amino acids overlap, covering the complete protein coding sequence (aa 5–1273) of the surface or spike glycoprotein (“S”, 130-127-953, Miltenyi Biotec) of SARS Coronavirus 2 (GenBank MN908947.3, Protein QHD43416.1), as well as a mixture of peptides covering the membrane glycoprotein (“M”, 30-126-702, Miltenyi Biotec) and the nucleocapsid phosphoprotein (“N”, 130-126-698, Miltenyi Biotec) protein of SARS-CoV-2. Additional wells included a positive control (CytoStim™, Miltenyi Biotec) and an unstimulated control, in which sterile water instead of the PepTivator pool was added. Except the positive control, all conditions were done in duplicates.

### Flow cytometry

After peptide stimulation for 16 hours, PBMC were stained with the reagents contained in the SARS-CoV-2 T cell analysis kit (PBMC), human plus anti-CD137-PE-Vio 615 (Miltenyi Biotec; see **Supplementary Table 2**) according to manufacturer’s recommendations and analyzed by flow cytometry.

For analysis of different T cell subsets, 2.5 x 10^6^ PBMCs were stained with viability stain LIVE/DEAD™ Fixable Blue Dead Cell Stain Kit, for UV excitation (L23105, Thermo Fisher) for 30 min. at 4°C. Afterwards, Fc receptors were blocked by incubating the cells with Human TruStain FcX™ (422302, Biolegend, RRID: AB_2818986) for 15 min. at 4°C. Subsequently, surface markers were stained for 15 min. at 4°C. Fixation and permeabilization were performed with Foxp3 staining buffer set (130-093-142, Miltenyi Biotec). Next, intracellular proteins Ki-67 and Foxp3 were stained for 30 min. at 4°C. Antibody dilutions are presented in **Supplementary Table 3**. Flow cytometry measurement was performed using a Cytoflex LX (Beckman Coulter, RRID: SCR_019627). Flow cytometry data were analyzed with FlowJo™ (Becton Dickinson & Company, version 10.8.0, RRID: SCR_008520) using either smoothing or large dot plot function for representation purpose. Barnes-Hut algorithm was implemented to generate t-Distributed Stochastic Neighbor Embedding (t-SNE) plots with FlowJo™ for high-dimensional data visualization.

### Statistics

Mann-Whitney or Kruskal–Wallis one-way ANOVA tests were performed to calculate statistical significance using Prism (GraphPad Software v9.2.0, San Diego, CA. RRID: SCR_002798). Antibody titers were compared by using two-tailed Welch´s test. The data were imported in R program (v3.6.3). Correlation analysis was performed using “rcorr” function of Hmisc package (v4.4-0) and visualized by “ggscatter” function of ggplot2-based ggpubr package (v0.2.5). Spearman method was used to compute the correlation coefficient (R) and p-value ≤ 0.05 was considered as significant correlation.

## Results

### Cohort characteristics

**Figure 1:**
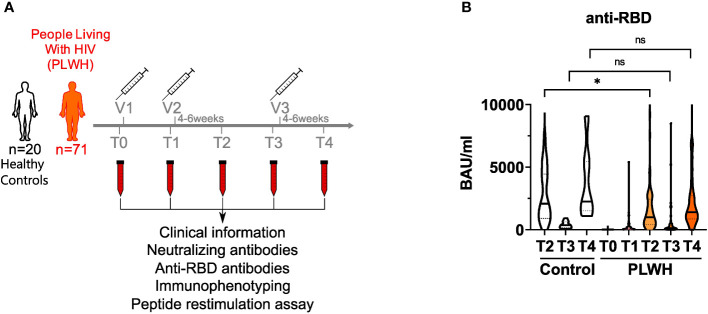
anti-RBD titers in vaccinated PLWH and HIV-negative controls. **(A)** Scheme showing the two groups of the study - control (n = 20) and people living with HIV (PLWH; n = 71) - and the time points of blood sampling (T0, T1, T2, T3 and T4) and vaccine inoculations (V1, V2, V3). **(B)** anti-RBD antibodies were determined by ELISA. Values are given as Binding Antibody Units (BAU) per ml. (PLWH T0, n = 71; PLWH T1, n = 61; PLWH T2, n = 68; PLWH T3, n = 47; PLWH T4, n=33; Control T2, n = 20; Control T3 & T4, n= 13). Statistical significance was calculated by two-tailed Welch´s test: *p < 0.05, ns, not significant.

We recruited a cohort of 71 people living with HIV (PLWH) receiving anti-retroviral therapy (ART) (mean age = 46.1 ± 10.9; 62 male and 9 female) and 20 HIV-negative donors (controls; mean age = 39.4 ± 11.9; 8 male and 12 female) to analyze humoral and cellular immune responses after two doses of vaccination. All participants received the BNT162b2 vaccine (Pfizer-BioNTech) apart from two participants of the control group, who got mRNA-1273 (Moderna) on the third vaccination time point. Except three participants, all of the PLWH cohort had a CD4 count higher than 200 cells/μl before and 3 months after vaccination (mean CD4 count: 756.4 cells/mm^3^; range: 79 – 1562 cells/mm^3^). None in the PLWH cohort reported a confirmed SARS-CoV-2 infection during the sampling period. One participant of the control group had a confirmed SARS-CoV-2 infection and got a BNT162b2 vaccination six months after the infection as recommended by the German Robert Koch Institute (RKI, Berlin). Since the responses of this donor were in the range of other HIV-negative donors, we did not exclude these values. HIV viral load was >30 copies/ml (range: 58-1573 copies/ml) in 6 participants at time of first vaccination. All but one (191 copies/ml) declined during follow up below detection level. The clinical data are summarized in **Supplementary Table 1**. Blood samples were drawn immediately before the first (T0) and second (T1) vaccination, four to six weeks after the second vaccination (T2), immediately before the third (T3) and four to six weeks after the third vaccination (T4) (**Figure 1A**).

### Reduced humoral immune responses in PLWH upon SARS-CoV-2 vaccination

During SARS-CoV-2 infection, most of the antibody response is directed against the receptor-binding domain (RBD) of the SARS-CoV-2 spike protein and serum levels of anti-RBD antibodies correlate well with the humoral immune response upon vaccination as well as protection against SARS-CoV-2 (6, 46). Therefore, we quantified anti-RBD antibodies in the plasma of study participants by ELISA. Notably, two donors in the PLWH group had detectable antibody titers already at T0 suggesting that they had a previous asymptomatic SARS-CoV-2 infection (**Figure 1B** and **Supplementary Table 4**). After the first vaccination, antibody titers did not rise significantly in PLWH (**Figure 1B**). After two doses of vaccine administration, antibody titers of PLWH were reduced (GMT: 838.42 BAU/ml; 95% confidence interval: 430.79-1246.05) compared to the HIV-negative control group (GMT: 1841.94 BAU/ml; 95% confidence interval: 850.35-2833.53) (**Figure 1B**). Although we did not have access to T1 samples in the control group, we could observe a 5-6 times increase in antibody titers in PLWH between the first and the second vaccination, while other studies reported 10-20 times titer increase in HIV-negative individuals (17, 47). Nevertheless, vaccination induced anti-RBD antibody titers in PLWH and additional doses of vaccination further increased these titers (**Figure 1B**). During the time between the second and third vaccination (time points T2 and T3), antibody titers dropped significantly in PLWH, and were boosted again by the third vaccination (T3 vs. T4) (**Figure 1B**). Of note, the PLWH group (anti-RBD titer average: 2394 BAU/ml) had lower antibody titers against the RBD after the third vaccination compared to the control group (anti-RBD titer average: 3733 BAU/ml), although this was not statistically significant (**Figure 1B**).

**Figure 2:**
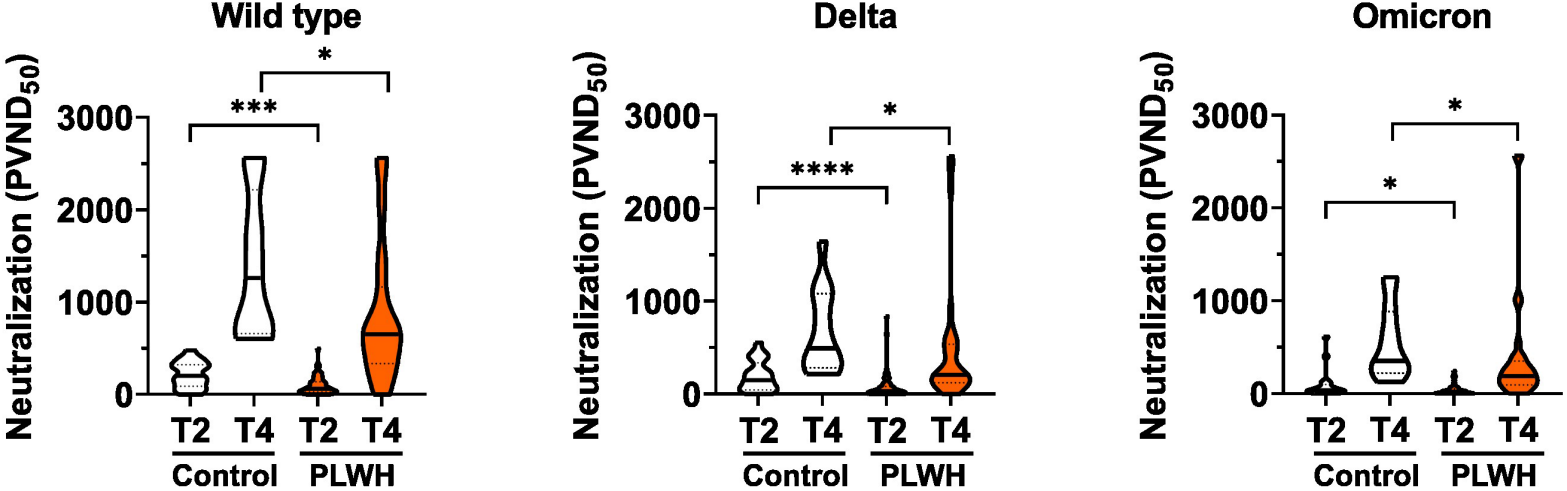
Neutralizing antibodies to SARS-CoV-2. Vero E6 cells were infected with the respective pseudoviruses in the presence of serial dilutions of sera. The pseudotype virus neutralization dose 50% (PVND50) is plotted. Neutralizing antibodies against the SARS-CoV-2 strains Wild type (PLWH T2, n = 70; Control T2, n = 20; Control T4, n = 13; PLWH T4, n = 33), Delta (PLWH T2, n = 65; Control T2, n = 20; Control T4, n = 13; PLWH T4, n = 33) and Omicron (PLWH T2, n = 70; Control T2, n = 20; Control T4, n = 13; PLWH T4, n = 33) were tested. Statistical significance was calculated by two-tailed Mann-Whitney test: *p < 0.05, ***p < 0.001, ****p < 0.0001.

Next, we analyzed the capacity of sera to neutralize SARS-CoV-2 infection. To this end, we employed an established pseudovirus assay (43) and determined the neutralization dose against the viral strain first reported in Wuhan, China (referred to hereafter as WT) as well as Delta and Omicron SARS-CoV-2 variants of concern. The amounts of neutralizing antibodies were reduced in PLWH compared to controls for all, WT, Delta and Omicron SARS-CoV-2 strains after the second and the third vaccination (**Figure 2**). We conclude that although SARS-CoV-2 vaccination induces antibody responses in PLWH the extent of the humoral immune response is reduced compared to HIV-negative controls.

### Altered cytotoxic T cell subsets and constitutive activation of CD8+ T cells in PLWH

Next, we analyzed cellular immune responses towards SARS-CoV-2 vaccination in PLWH and control groups. Here, multi-parameter flow cytometry was employed and the gating strategy is shown in **Supplementary Figure 1**. As expected, we detected no differences in the frequencies of total CD3+ T cells between PLWH and the control group (**Supplementary Figure 2A**), reduced frequencies of CD4+ T cells in PLWH (**Supplementary Figure 2B**), and increased frequencies of CD8+ T cells (**Supplementary Figure 2C**). Accordingly, the ratio of CD4 to CD8 T cells was significantly lower in PLWH compared to the control group (**Supplementary Figure 2D**).

Within the CD8+ compartment, the naïve CD8+ T cells (CD27+ CD45RA+ CCR7+) were reduced in PLWH compared to controls (**Supplementary Figure 3A**). Moreover, we detected increased frequencies of effector (E; CD27- CD45RA+ CCR7-; **Supplementary Figure 3B**) and effector memory CD8+ T cells (EM; CD27- CD45RA- CCR7-; **Supplementary Figure 3C**) as well as decreased frequencies of transitional memory CD8+ T cells (TM; CD27+ CD45RA- CCR7-; **Supplementary Figure 3D**) in PLWH. The observation that CD8+ T cells are less naïve and more in an activated and effector-like state in PLWH than in controls was also supported by unsupervised clustering using t-SNE for two representative donors (**Supplementary Figure 3E**). Thus, the phenotypic profiling of the CD8 T cell compartment suggests a high constitutive activation of these cells. In line with this notion, we detected increased frequencies of activated, i.e. CD137 expressing, CD8+ cells that co-expressed the inflammatory cytokines IFNγ and TNFα even in the absence of any antigenic peptide stimulation making it impossible to measure antigen-specific responses in the CD8 compartment (**Supplementary Figure 4** and **Supplementary Figures 5A, B**). Moreover, increased frequencies of CD8+ CD137+ IFNγ+ TNFα+ T cells were detected in PLWH upon stimulation with peptide pools derived from spike (S) protein as well as membrane plus nucleocapsid proteins (M+N) (**Supplementary Figures 5C, D**) suggesting that this response is unlikely to be related to the vaccination. Thus, in contrast to what has been reported for SARS-CoV-2 infection in PLWH (41), we were not able to measure antigen-specific CD8 T cell responses in our cohort. However, our data are in line with the activated phenotype described for CD8+ T cells in other studies (48).

### Comparable CD4+ T cells responses in PLWH and HIV-negative controls upon SARS-CoV-2 vaccination

Furthermore, we analyzed cellular responses of CD4+ T helper cells upon vaccination. Surprisingly, we detected decreasing frequencies of circulating follicular T helper (cTFH) cells, which are characterized by the expression of CXCR5 and PD-1, over the course of vaccination in PLWH (**Supplementary Figure 6A**). However, we did not observe any correlation between cTFH cells and antibody responses (neutralizing or anti-RBD; data not shown). Consistent with the situation observed for CD8+ T cells in PLWH, we found reduced frequencies of naïve Tcon cells and increased frequencies of terminally differentiated Tcon cells in PLWH after vaccination with two doses (**Supplementary Figures 6B, C**). Despite a similar tendency in these populations, the differences between the groups were not significant in T3 and T4 time points (**Supplementary Figures 6B, C**). Interestingly, we observed significant differences in effector memory T cells for all time points (**Supplementary Figure 6D**). Again, unsupervised clustering using t-SNE of two representative donors supported our findings that CD4+ T cells of PLWH are rather in an activated and differentiated state and that the naïve phenotype is reduced (**Supplementary Figure 6E**).

**Figure 3:**
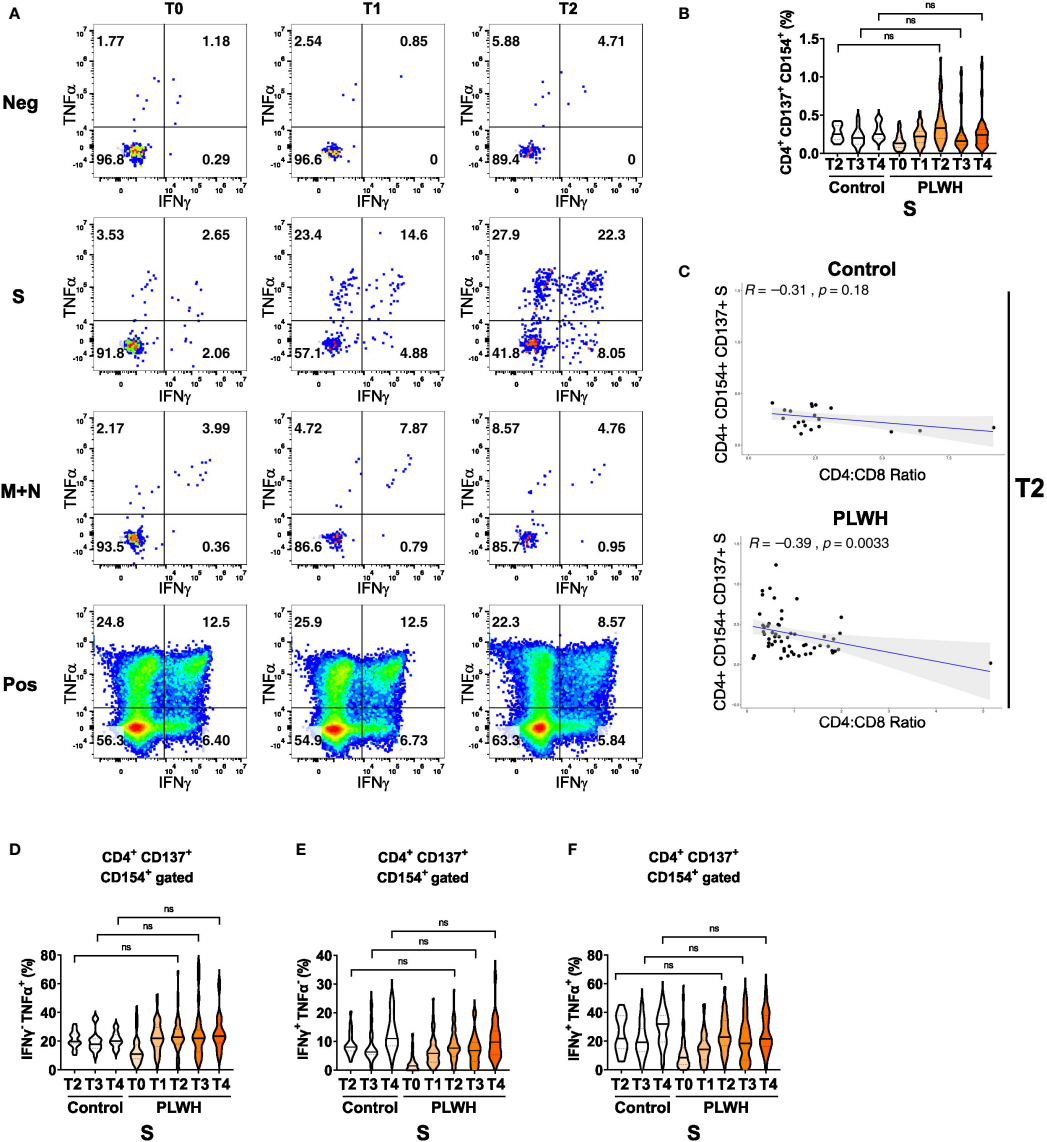
Antigen-dependent responses of CD4+ helper T cells in vaccinated PLWH and HIV-negative controls. **(A)** Representative dot plots of TNFα and IFNγ expression in activated CD4+ CD137+ CD154+ T cells of a PLWH donor stimulated with SARS-CoV-2 spike (S) glycoprotein peptide pool, or with membrane glycoprotein plus nucleocapsid phosphoprotein (M+N) peptide pool, or with CytoStim™ (Pos), or left untreated (Neg) before and after vaccination shots. **(B)** The violin plot represents frequency of activated, i.e. CD137+ CD154+, CD4+ T cells and **(C)** their correlation (spearman method) with CD4:CD8 ratio from control subjects (upper scatter plot) and PLWH (lower scatter plot). The violin plots represent **(D)** frequency of TNFα+ cells in activated CD4+ CD137+ CD154+ T cells, **(E)** frequency of IFNγ+ cells in activated CD4+ CD137+ CD154+ T cells and **(F)** frequency of TNFα+ IFNγ+ double-positive cells in activated CD4+ CD137+ CD154+ T cells at the indicated time points. Statistical significance was calculated by two-tailed Mann-Whitney test: ns, not significant (PLWH T0, n = 64-65; PLWH T1, n = 54; PLWH T2, n = 59-61; PLWH T3 & T4, n = 29; Control T2, n = 20; Control T3 &T4, n = 15).

Finally, we analyzed antigen-specific responses in CD4+ T helper cells. We detected only minimal cytokine responses in cultures without any peptide stimulation or in cultures stimulated with peptide pools derived from membrane (M) and nucleocapsid (N) proteins (**Figure 3A** and **Supplementary Figure 7A**). In contrast, polyclonal stimulation (positive control (Pos) with CytoStim™) resulted in strong responses at all time points (**Figure 3A**). Stimulation with spike (S) protein peptide pools resulted in upregulation of the activation markers CD137 and CD154 on CD4+ T cells of PLWH after the first and the second vaccination (**Figure 3B**). After a certain reduction of the two activation markers at time point T3, the third vaccination increased CD137 and CD154 expression again at time point T4 (**Figure 3B**). Of note, we did not observe statistically significant differences in the frequencies of activated CD154+ CD137+ CD4+ T cells upon S peptide re-stimulation between the two groups (**Figure 3B**). Surprisingly, we found a significant anti-correlation between S-peptide antigen-specific CD4 T cells expressing activation markers CD154+ CD137+ and the ratio of CD4 to CD8 in the PLWH cohort (R=-0.39; p-value=0.0033; **Figure 3C** lower scatter plot), which was insignificant in the HIV-negative control group (R=-0.31; p-value=0.18; **Figure 3C** upper scatter plot). Thus, low CD4+ relative to CD8+ T cell counts are surprisingly associated with better activation of CD4+ T cells.

We then measured IFNγ and TNFα expression in the CD154 and CD137 co-expressing CD4+ T cells and found a robust increase in single (IFNγ or TNFα) or double (IFNγ and TNFα) cytokine-producing cells in PLWH after the first, second and third vaccination (**Figures 3D-F**). Importantly, the expression of cytokines exhibited no significant differences between PLWH and the control group. Furthermore, comparable findings were obtained when activated CD4+ T cells that only expressed the activation marker CD154 were analyzed for S-peptide stimulation (**Supplementary Figures 7B, D**) Therefore, despite the known reduced frequency of CD4+ T helper cells in PLWH, the antigen-specific responses in the CD4 compartment are comparable between PLWH and control groups.

### Correlation analysis of immune response parameters in vaccinated PLWH and HIV-negative controls

**Figure 4:**
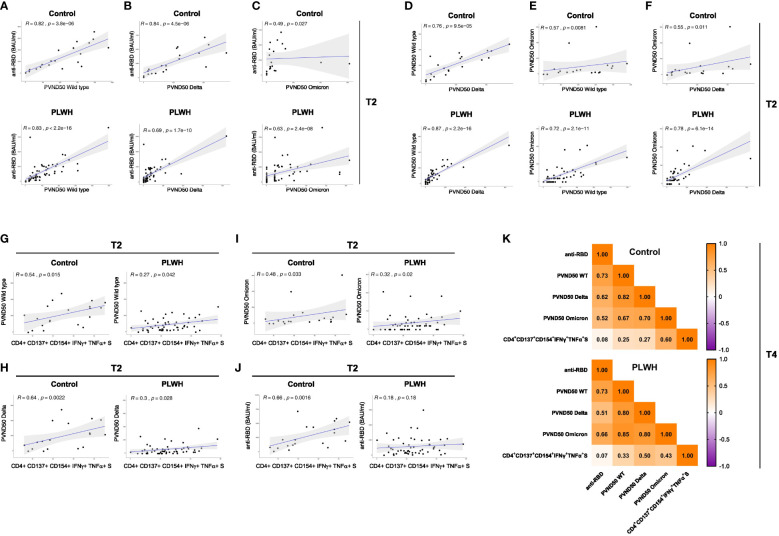
Correlation analysis of immune response parameters in vaccinated PLWH and HIV-negative controls. **(A-C)** The scatter plots represent correlation analyses between anti-RBD titers and neutralizing capacity against the SARS-CoV-2 WT, Delta and Omicron strains for control and PLWH groups. **(D-F)** The scatter plots represent correlation analyses among the neutralizing capacities against the indicated SARS-CoV-2 strains for control and PLWH groups. **(G-J)** CD4+ CD137+ CD154+ T cells expressing both IFNγ+ and TNFα+ upon spike protein-derived peptide pool stimulation were correlated using spearman method for time point T2. The scatter plots represent correlation between CD4+ CD137+ CD154+ IFNγ+ TNFα+ T cells and neutralizing capacity against WT strain of SARS-CoV-2 **(G)**, Delta variant of SARS-CoV-2 **(H)**, Omicron variant of SARS-CoV-2 **(I)** and against anti-RBD titers **(J)** on the left side for controls, while scatter plots on the right side are for PLWH cohort. Black dots represent the values of the individual donors. The regression line is indicated in blue, while the gray area represents the 95% confidence interval region. In the graphs, R indicates the correlation coefficient and p stands for p-value of statistical significance. **(K)** Correlation matrix of the indicated parameters obtained at time point T4. Spearman method was used for correlation analyses. The values of the correlation coefficient R is given.

After performing correlation analysis of the parameters obtained after the second vaccination (T2) of the study, we found a statistically significant correlation between anti-RBD antibody response and neutralization capacity against WT SARS-CoV-2 strain in HIV-negative participants (R=0.82, p=3.8e-06) as well as PLWH cohorts (R=0.83, p<2.2e-16) (**Figure 4A**). However, the correlation analyses for anti-RBD antibody and neutralization against SARS-CoV-2 Delta strain were remarkably decreased in PLWH patients (R=0.69, p=1.7e-10) and controls (R=0.84, p=4.5e-06) (**Figure 4B**). No significant correlation was found for anti-RBD antibody and neutralization against the Omicron variant in the control group (**Figure 4C**). The neutralization capacity against WT, Delta and Omicron strains of SARS-CoV-2 correlated better in PLWH patients compared to HIV-negative individuals (**Figures 4D–F**), which might be due to the higher number of participants in the PLWH cohort.

Regarding antigen-specific T cell responses, the correlation analyses at the T2 time point in the control participants showed a significant positive correlation between S-peptide antigen-specific CD4+ CD154+ CD137+ T cells producing IFNγ and TNFα cytokines and neutralizing capacity against SARS-CoV-2 WT strain (R=0.54; p-value=0.015; **Figure 4G** left graph), Delta variant (R=0.64, p-value=0.0022; **Figure 4H** left graph), Omicron variant (R=0.48, p-value=0.03; **Figure 4I** left graph) and with anti-RBD titer (R=0.66; p-value=0.0016; **Figure 4J** left graph). Interestingly, the correlation value R of these comparisons in PLWH cohort exhibited a significant weaker association (**Figures 4G–I** right graphs) and a non-significant correlation for anti-RBD (R=0.18; p-value=0.18, **Figure 4J** right graph). Moreover, we found comparable correlations between TNFα- and IFNγ-producing CD4+ CD154+ cells with neutralization against Delta and Omicron variants (**Supplementary Figures 7E, F**).

We then performed correlation analysis of the parameters obtained at time point T4 (after the third vaccination). Similar to time point T2, we found comparable correlations between anti-RBD antibody response and neutralization capacity against WT SARS-CoV-2 strain in HIV-negative participants and in PLWH after the third vaccination (**Figure 4K**). In the same line, PLWH presented weaker correlation for anti-RBD antibody and neutralization against SARS-CoV-2 Delta than the control group (**Figure 4K**). Similar to T2, the control group had a weak correlation for anti-RBD antibody and neutralization against the Omicron variant (**Figure 4K**). Except for the case between the neutralization capacity against the Delta strain, which showed comparable results, the correlations for the neutralization capacity against WT and Omicron were stronger in PLWH compared to control group after the third vaccination (**Figure 4K**). Of note, there was practically no correlation for anti-RBD and S-peptide antigen-specific CD4+ CD154+ CD137+ T cells producing IFNγ and TNFα cytokines in both groups (**Figure 4K**). Although weak in general, the latter parameter showed stronger positive correlation with neutralization capacity against WT and Delta strains in PLWH (**Figure 4K**). On the contrary, the correlation between S-peptide antigen-specific CD4+ CD154+ CD137+ T cells producing IFNγ and TNFα cytokines and neutralization capacity against Omicron strain was stronger in the control group than in PLWH (**Figure 4K**).

In conclusion, while the cellular immune response by CD4+ T cells is surprisingly comparable to the control group, the humoral response mediated by antibodies is reduced suggesting that PLWH might be less protected from COVID-19 by vaccination.

## Discussion

The occurrence of SARS-CoV-2 in the human population in late 2019 led to a pandemic that is causing a major health care burden worldwide. In an effort to cope with the pandemic, several vaccines have been developed that reduce the risk to suffer from severe COVID-19 and of mortality by inducing humoral and cellular immune responses [reviewed in (49)]. Particularly, the novel mRNA vaccines show high efficacy and are well tolerated (16, 17, 50). However, COVID-19 vaccines may induce less efficient immune responses in the context of immunosuppression. Accordingly, antibody responses to spike protein in people with inborn errors of immunity were reduced compared to controls when CD3+ T cells counts were lower than 1000 cells/ml or CD19+ B cell counts were lower than 100 cells/ml (51). Moreover, impaired antibody, B cell and T cell responses have been reported for patients receiving kidney transplantation receiving COVID-19 mRNA vaccination (52–56).

Here, we addressed the question whether COVID-19 mRNA vaccines are able to mount immune responses in PLWH, since HIV can cause acquired immunodeficiency. To do so, we analyzed humoral, i.e. antibody, and cellular immune responses in PLWH over the time course of vaccination and compared the responses after three doses of vaccine with fully vaccinated HIV-negative controls. We detected anti-RBD antibodies and neutralizing antibodies against the original strain (WT) of SARS-CoV-2 as well as against the Delta and Omicron variants in PLWH albeit at lower levels than in the control group. Especially the neutralizing antibody titers against the Delta and Omicron variants were low in PLWH and the general antibody titers correlated less well with neutralizing antibodies against Delta as compared to the correlation of anti-RBD antibodies versus neutralizing antibodies against the SARS-CoV-2 WT strain. This might indicate an improved immune evasion of the Delta variant and raises concerns about the protection that two doses of mRNA vaccine mount in PLWH against current and upcoming variants of concern of SARS-CoV-2. Our results on the humoral immune response are in agreement with other reports that reported on immunogenicity and safety of COVID-19 vaccines (57–59). In contrast, similar antibody responses were reported in a PLWH cohort that received an inactivated SARS-CoV-2 vaccine (60).

It has been previously described that a persistent anti-RBD antibody response is associated with a reduced risk of COVID-19 reinfection (61). Interestingly, we did not observe such an association in PLWH. In the clinical follow-up, we identified eight patients from our PLWH cohort, who got COVID-19 breakthrough infection after the second vaccination (time point T2) and six after the third vaccination (time point T4). However, these breakthrough infections occurred in patients with low and high anti-RBD antibody levels alike. Thus, it may be that anti-RBD antibody levels might not be a good parameter to assess the COVID-19 infection risk in PLWH. Since we observed significant differences in anti-RBD antibody levels on the T2 and T4 time points between PLWH and HIV-negative controls (**Figure 1**) and both groups are not fully matched for gender, we performed additional linear regression analysis to test if the observed differences are due to the gender composition in both cohorts (data not shown). Although our PLWH and HIV-negative control cohorts consist of 87% and 40% male, respectively, we did not find a significant association of gender and anti-RBD titers by the end of our longitudinal study. There was only one time point, namely T2, at which we found a statistically significant difference (p-value 0.0285) in the average anti-RBD antibody titers between males (mean value 1350.8 BAU/ml) and females (mean value 2711.2 BAU/ml) from the PLWH cohort. Of note, the control group did not show a significant difference at the T2 time point. Taken together, this implies that the imbalanced gender distribution in the two groups has no direct biological impact on the outcome of the study. Nevertheless, it would be interesting to address gender bias in a larger cohort of study participants as HIV is more prevalent in males than females.

Next to antibody titers, we also analyzed cellular immune responses in PLWH upon COVID-19 vaccination by multi-parametric flow cytometry. As expected (62), we detected a reduced CD4+ to CD8+ T cell ratio and CD8+ T cells exhibited a more activated phenotype. A reduced naïve CD8 compartment and a higher prevalence of effector, effector memory and transitional memory CD8+ T cells was not only observed by conventional flow cytometry analysis but also by unsupervised t-SNE clustering. Additionally, the activated phenotype of CD8+ T cells prevented the analysis of antigen-specific CD8+ T cell responses since these cells expressed the type I immunity signature cytokines IFNγ and TNFα already in the absence of any stimulation in our *in vitro* cultures.

A higher activation and a reduced naïve compartment in PLWH were also observed for CD4+ T cells, again by conventional flow cytometry analysis and unsupervised clustering using t-SNE. Nevertheless, we were able to analyze antigen-specific CD4+ T cell responses since the expression of cytokines in the absence of any re-stimulating peptides was negligible. Of note, we detected higher responses to the peptide pool containing M and N derived peptides in PLWH after full vaccination as compared to the control group (**Supplementary Figure 7A**). One explanation could be that a few donors of the PLWH cohort contracted an asymptomatic SARS-CoV-2 infection before start of the vaccination regime. Although no case of symptomatic COVID-19 was reported to us during sampling of the PLWH cohort, we indeed detected anti-RBD antibodies in two PLWH donors suggesting asymptomatic SARS-CoV-2 infections. An alternative explanation might be that these responses are due to previous exposures to other human corona viruses, which can cause common cold symptoms and might share common epitopes with SARS-CoV-2. Thirdly, and not mutually exclusive to the first two potential explanations, the anti-retroviral therapy (ART) that our cohort participants received might enhance immune responses against SARS-CoV-2 as has been reported by epidemiological studies (63–65). More importantly, we detected an increase in the expression of the activation markers CD137 and CD154 as well as in the expression of the cytokines IFNγ and TNFα by CD4+ T cells over the time course of vaccination. Notably, the expression of these markers and the frequency of multifunctional T cells, which express both IFNγ and TNFα, was similar in PLWH and in the control group. Thus, despite the reduced CD4+ T cell count in the peripheral blood of PLWH, the CD4+ cellular response to COVID-19 vaccination is preserved. Furthermore, we found a positive correlation between the frequency of multifunctional CD4+ T cells (CD4+ CD137+ CD154+ IFNγ+ and TNFα+) and neutralizing antibody responses in PLWH. However, in comparison to the one in the controls, the correlation was less pronounced after two vaccinations for all SARS-CoV-2 strains and remained lower for Omicron after the booster vaccination. Moreover, the correlation for multifunctional CD4+ T cells and anti-RBD antibodies that was observed in the control group at T2 was absent in PLWH.

In addition, we found that the CD4 to CD8 ratio negatively correlated with T cell activation, as detected by the presence of CD4+ CD137+ CD154+ cells, in response to antigens derived from the spike protein in PLWH. No such significant correlation was found in the control group. Therefore, a reduction in CD4+ relative to CD8+ T cells appears to result in better T cell activation in PLWH. Moreover, the CD4 to CD8 ratio might be a predictive biomarker for the effectiveness of COVID-19 vaccines in PLWH. Similar observations have been made for hepatitis B virus and Yellow Fever virus vaccines (66, 67).

The diminished antibody responses detected in our PLWH cohort are in line with reports of reduced antibody titers upon vaccination of PLWH with an influenza A virus vaccine (68–70) or the mycobacterial vaccine strain BCG (71). However, while some of these studies also reported reduced T cell responses (68, 69), we found similar CD4+ T cell responses in PLWH vaccinated with an COVID-19 mRNA vaccine. Whether these differences are due to differences in the viral antigen or due to the different vaccine formulation remains to be tested in future studies.

In line with our study, a recent work investigating the immunogenicity of mRNA-based vaccines in PLWH showed the importance of repeated vaccination to boost the immunity against SARS-CoV-2 in this group (72). Interestingly, the authors could also observe that, while humoral responses were more potent after each vaccination, the intensity of the cellular response remained stable after the second vaccination (72). While the CD4+ T cell responses towards spike protein-derived peptides are promising, the reduced antibody responses confirm that two and even three doses of mRNA vaccine may be insufficient to protect PLWH from COVID-19. Recent evidence suggests that a booster by a third and fourth dose of vaccine not only enhances antibody levels, which might decrease over time, but also broadens the antibody-mediated immunity and can protect against variants of concern (73–76). Therefore, additional booster vaccinations are required for PLWH to maintain or to reach full protection from COVID-19.

*Limitations of the study:* The PLWH participants in our study received well-adjusted anti-retroviral therapy (ART) and had CD4+ T cells counts higher than 200 cells/μl. Whether immune responses upon COVID-19 vaccination correlate with T cell numbers in the peripheral blood awaits the analysis of a larger cohort with more diverse T cell counts. A further limitation is the lack of control samples before the second vaccination (T0 and T1), which were unavailable since participants of the control group were already vaccinated before the beginning of this study. Moreover, we did not test directly for vaccine safety or efficacy and gender bias since this would be beyond the scope of this study. Nevertheless, no gross adverse effects were reported by the participants of our PLWH cohort. Furthermore, our results suggest that vaccination of PLWH with COVID-19 mRNA vaccines might elicit cellular and at least partial humoral immune protection.

## Data availability statement

The original contributions presented in the study are included in the article/**Supplementary Materials**. Further inquiries can be directed to the corresponding author.

## Ethics statement

The studies involving human participants were reviewed and approved by the local ethics committee of the Ruhr-University Bochum (21-7351 and 20-6953-bio). The patients/participants provided their written informed consent to participate in this study.

## Author contributions

AP, AS, AS-R and NB acquired patient samples. CB, CP-S, AS, CM, EB, PB, SB, SS and DU performed experiments. CB, CP-S, AS, JB, SP and IS analyzed data. CB, CP-S, JB and IS wrote the draft of the manuscript. All authors read and approved the final version of the manuscript. AP, SF, OO, AR-S, CW, SP and IS designed the study. AP and IS supervised the study. All authors contributed to the article and approved the submitted version.

## Funding

This project was financially supported by the State of North Rhine‐Westphalia, Germany, as part of the research grant “SARS-CoV-2 specific T-cell diagnostic”, project no. MIL-1-1.

## Acknowledgments

We would like to thank all blood donors who participated in this study. We are grateful to all members of the WIR - Walk In Ruhr, Center for Sexual Health and Medicine, Bochum, Germany, for supporting this study. We thank Dr. Gert Zimmer, Institute for Virology und Immunology, Switzerland and Department of Infectious Diseases and Pathobiology (DIP), Vetsuisse Faculty, University of Bern, Switzerland as well as Dr. Stefan Pöhlmann and Markus Hoffmann, Infection Biology Unit, German Primate Center - Leibniz Institute for Primate Research, Göttingen, Germany, Faculty of Biology and Psychology, Georg-August-University Göttingen, Göttingen, Germany, for providing the WT, Delta and Omicron plasmids. We thank Dr. Sabrina Mühlen for critically reading the manuscript. We thank Miltenyi Biotec and especially Dr. Marc Schuster and Dr. Bahram Kasmapour for support with antigen-specific T cell assays. We acknowledge support by the Open Access Publication Funds of the Ruhr-Universität Bochum.

## Conflict of interest

The authors declare that the research was conducted in the absence of any commercial or financial relationships that could be construed as a potential conflict of interest.

## Publisher’s note

All claims expressed in this article are solely those of the authors and do not necessarily represent those of their affiliated organizations, or those of the publisher, the editors and the reviewers. Any product that may be evaluated in this article, or claim that may be made by its manufacturer, is not guaranteed or endorsed by the publisher.
