# Supplementary material for: Impact of SARS-CoV-2 vaccination on systemic immune responses in people living with HIV
Source: Front Immunol. 2022 Dec 2;13:1049070. doi: 10.3389/fimmu.2022.1049070 (PMC9755486; doi:10.3389/fimmu.2022.1049070)
Supplement: Supplementary file 1 [file DataSheet_1.pdf]

Supplementary Material

Supplementary Table 1

	Control	PLWH
**n**	20	71
**Age (Mean ± SD)**	39.4 ± 11.9	46.1 ± 10.9
**Sex (F/M)**	12/8	9/62
**HIV**	-	+
**Antiretroviral therapy**	-	+

Supplementary Table 2

Specificity	Clone	Fluorochrome	Dilution	RRID
CD3	REA613	APC	1:50	AB_2726238
CD4	REA623	VioBright B515	1:50	AB_2726921
CD8	REA734	VioGreen	1:50	AB_2659241
CD14	REA599	VioBlue	1:50	AB_2655055
CD20	REA780	VioBlue	1:50	AB_2656068
CD137 (4-1BB)	REA765	PE-Vio 615	1:50	AB_2654990
CD154 (CD40L)	REA238	APC-Vio 770	1:50	AB_2904741
IFN-γ	REA600	PE	1:50	AB_2733717
TNF-α	REA656	PE-Vio 770	1:50	AB_2905446
Viobility Fixable Dye	-	405/452	1:100	-

Supplementary Table 3

Specificity	Clone	Fluorochrome	Cat. number	Company	Dilution	RRID
CD3	UCHT1	Brilliant Violet 510	300448	Biolegend	1:100	AB_2563468
CD4	OKT4	PerCP-Cy5.5	344608	Biolegend	1:500	AB_1953236
CD8	RPA-T8	APC- Fire 750	344746	Biolegend	1:500	AB_2572095
CD19	HIB19	AlexaFluor 700	302225	Biolegend	1:500	AB_493750
CD25	BC96	Brilliant Violet 421	302630	Biolegend	1:200	AB_11126749
CD27	O323	Brilliant Violet 785	302831	Biolegend	1:100	AB_11219185
CD45RA	HI100	PerCP	304155	Biolegend	1:100	AB_2616996
CD95	DX2	Brilliant Violet 650	305642	Biolegend	1:100	AB_2632622
CD107a	H4A3	Brilliant Violet 711	328639	Biolegend	1:200	AB_2565839
CD127	HIL-7R-M21	BUV737	612794	BD Biosciences	1:20	AB_2870121
CD185 (CXCR5)	J252D4	AlexaFluor 488	356911	Biolegend	1:100	AB_2561893
CD197 (CCR7)	G043H7	APC	353213	Biolegend	1:100	AB_10915474
CD279 (PD-1)	EH12.2H7	PE-Dazzle 594	329939	Biolegend	1:20	AB_2563658
Foxp3	206D	PE	320107	Biolegend	1:20	AB_492987
Ki-67	Ki-67	PE-Cy7	350525	Biolegend	1:1000	AB_2562871

Supplementary Table 4 Immune response main values of donors with detectable anti-RBD titers at time point T0: Donor IDs 12 and 28. PVND50: pseudotype virus neutralization dose 50%. (S): SARS-CoV-2 Spike protein peptide pool. NA: Not available.

**Donor ID**	**Time point**	**PVND50 WT**	**PVND50 Delta**	**PVND50 Omicron**	**anti-RBD (BAU/ml)**	**TNF^+ IFN^+ of $CD4^+$ $CD154^+$ (S)**	**TNF^+ IFN^+ of $CD4^+$ $CD154^+$ $CD137^+$ (S)**
12	T0	NA	NA	NA	304.49	0	0
28	T0	NA	NA	NA	59.99	0.53	3.1
Mean PLWH T0					5.69	2.92	11.79
Max PLHW T0					304.49	31.5	58.8
12	T1	NA	NA	NA	336.85	0	0
28	T1	NA	NA	NA	NA	NA	NA
Mean PLWH T1					183.08	5.62	15.41
Max PLHW T1					1364.98	27.45	45.9
12	T2	53.41	20	0	2544.97	0	0
28	T2	62.27	115.5	23.31	277.51	3.14	12.95
Mean PLWH T2		92.47	68.72	46.81	1515.67	10.96	24.80
Max PLHW T2		495.3	836.2	699.9	9184.86	27.75	57.8
12	T3	NA	NA	NA	483.22	0.00%	0.00%
28	T3	NA	NA	NA	NA	NA	NA
Mean PLWH T3					266.47	12%	21%
Max PLHW T3					2127.65	43%	51%
12	T4	1441	569.4	983.8	6782.11	6.00%	8.03%
28	T4	NA	NA	NA	NA	NA	NA
Mean PLWH T4		895.93	491.53	414.83	2463.27	16%	26%
Max PLHW T4		2560	2560	2560	14403.58	32%	54%

Supplementary Figures

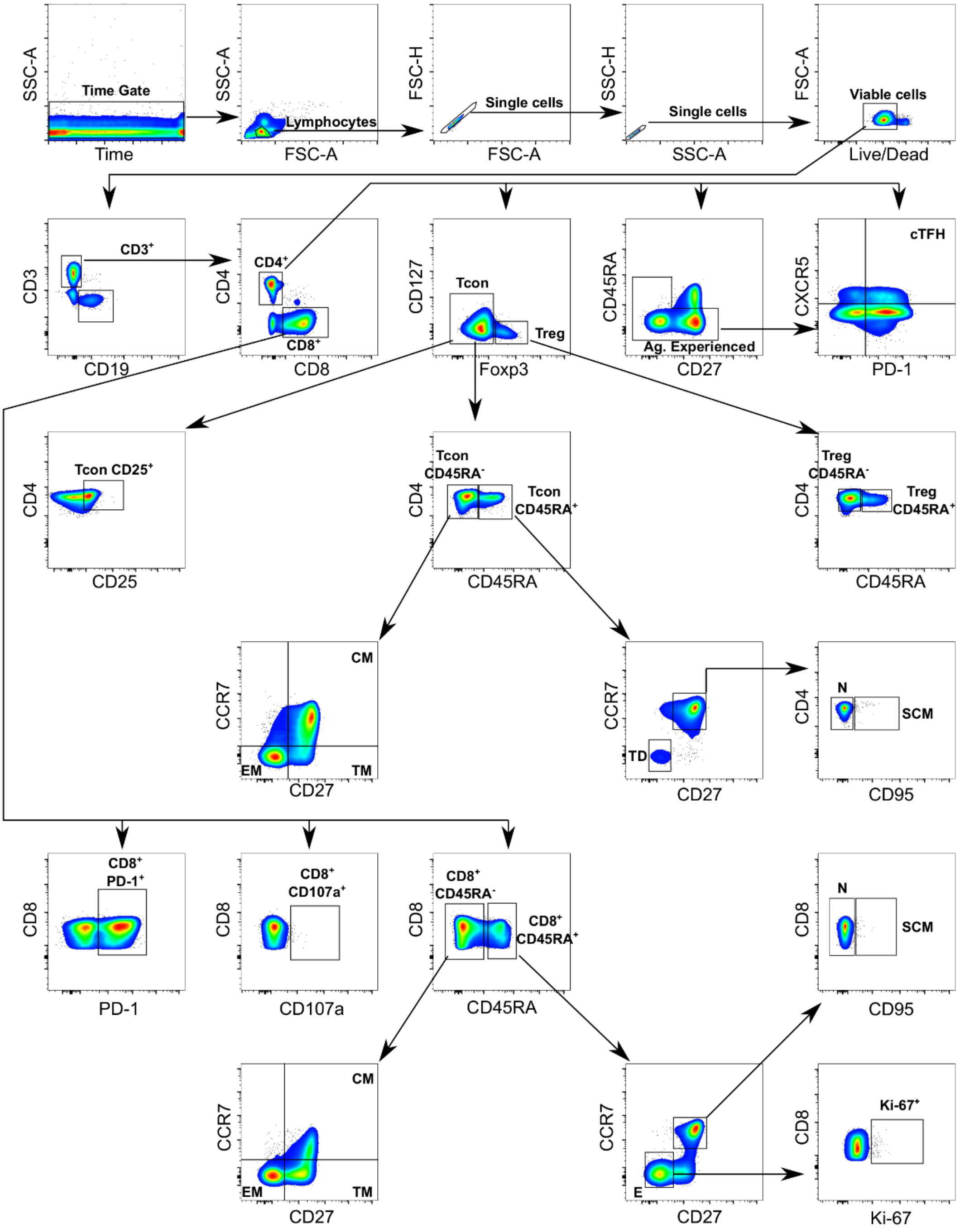

Supplementary Figure 1 Gating strategy for analysis of T cell subsets via flow cytometry. Abbreviations used: cTFH (circulating follicular T helper cells), N (Naïve), SCM (Stem cell-like memory), CM (Central memory), TM (Transitional memory), EM (Effector memory), E (Effector), TD (Terminally differentiated).

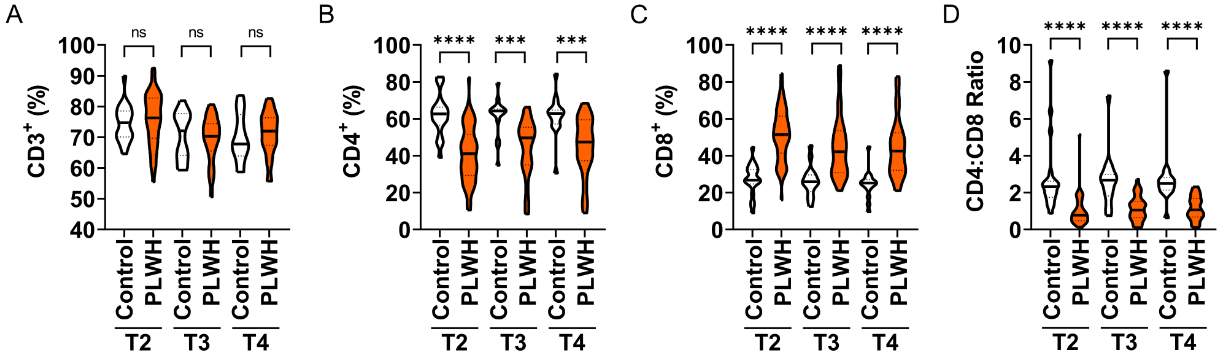

Supplementary Figure 2 Frequency of lymphocyte lineages in vaccinated PLWH and HIV^{-} controls. Frequencies of CD3+ **(A)**, CD4+ **(B)**, CD8+ **(C)** cells at the indicated time point. **(D)** The CD4:CD8 ratio is plotted for the control and PLWH groups at the indicated time points. Statistical significance was calculated by two-tailed Mann-Whitney test: ***p < 0.001, ****p < 0.0001, ns= not significant (PLWH T2, n= 64; PLWH T3, n= 29; PLWH T4, n= 15; Control T2, n= 20; Control T3 and T4, n= 15).

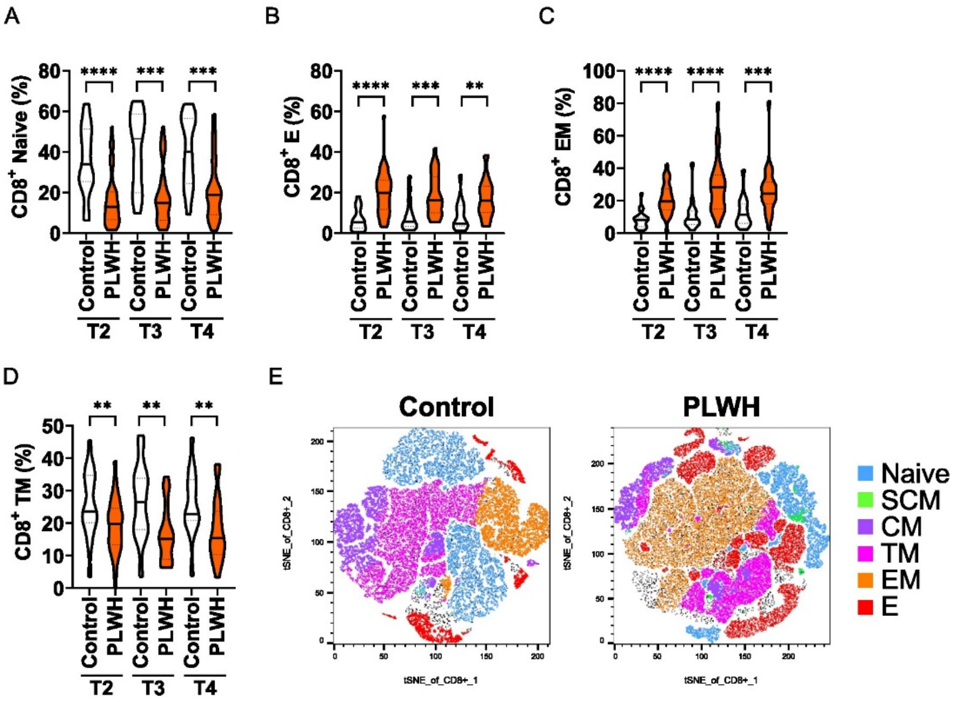

Supplementary Figure 3 Phenotype and antigen-dependent responses of CD8+ cytotoxic T cells in vaccinated PLWH and HIV-negative controls. Frequencies of Naïve **(A)**, Effector (E) **(B)**, Effector-Memory (EM) **(C)** and transitionally memory (TM) **(D)** CD8+ cells at the indicated time points. **(E)** Representative t-SNE plots of the distribution of naïve, stem cell-like memory (SCM), central memory (CM), transitional memory (TM), effector memory (EM) and effector (E) CD8+ T cells for control and PLWH groups. Statistical significance was calculated by two-tailed Mann-Whitney test: $^{**}p < 0.01$, $^{***}p < 0.001$, $^{****}p < 0.0001$ (PLWH T2, n= 64; PLWH T3 & T4, n= 29; Control T2, n= 20, Control T3 & T4, n= 15).

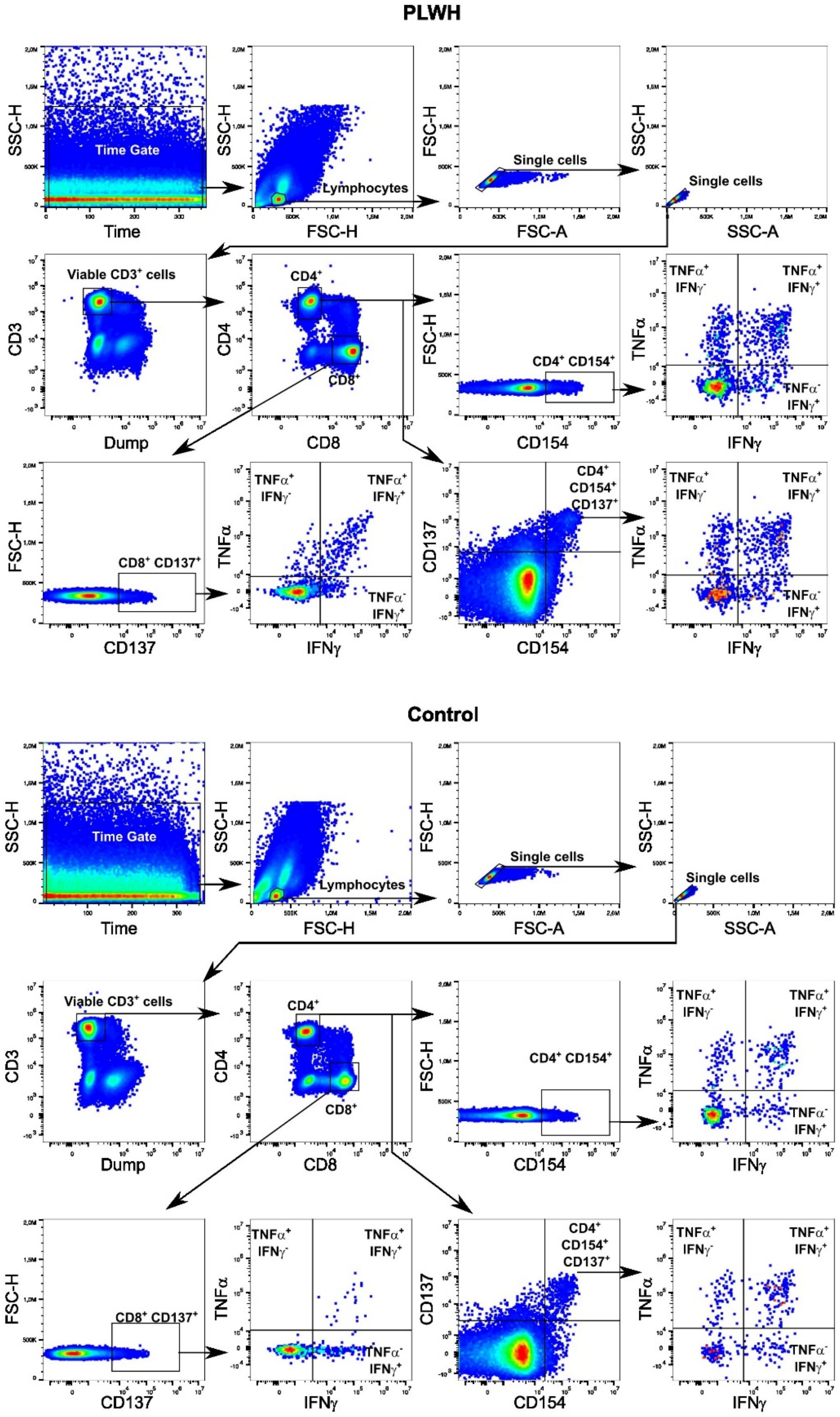

Supplementary Figure 4 Gating strategy for analysis of antigen-specific T cells via flow cytometry. Representative dot plots for PLWH group (top) and Control group (bottom).

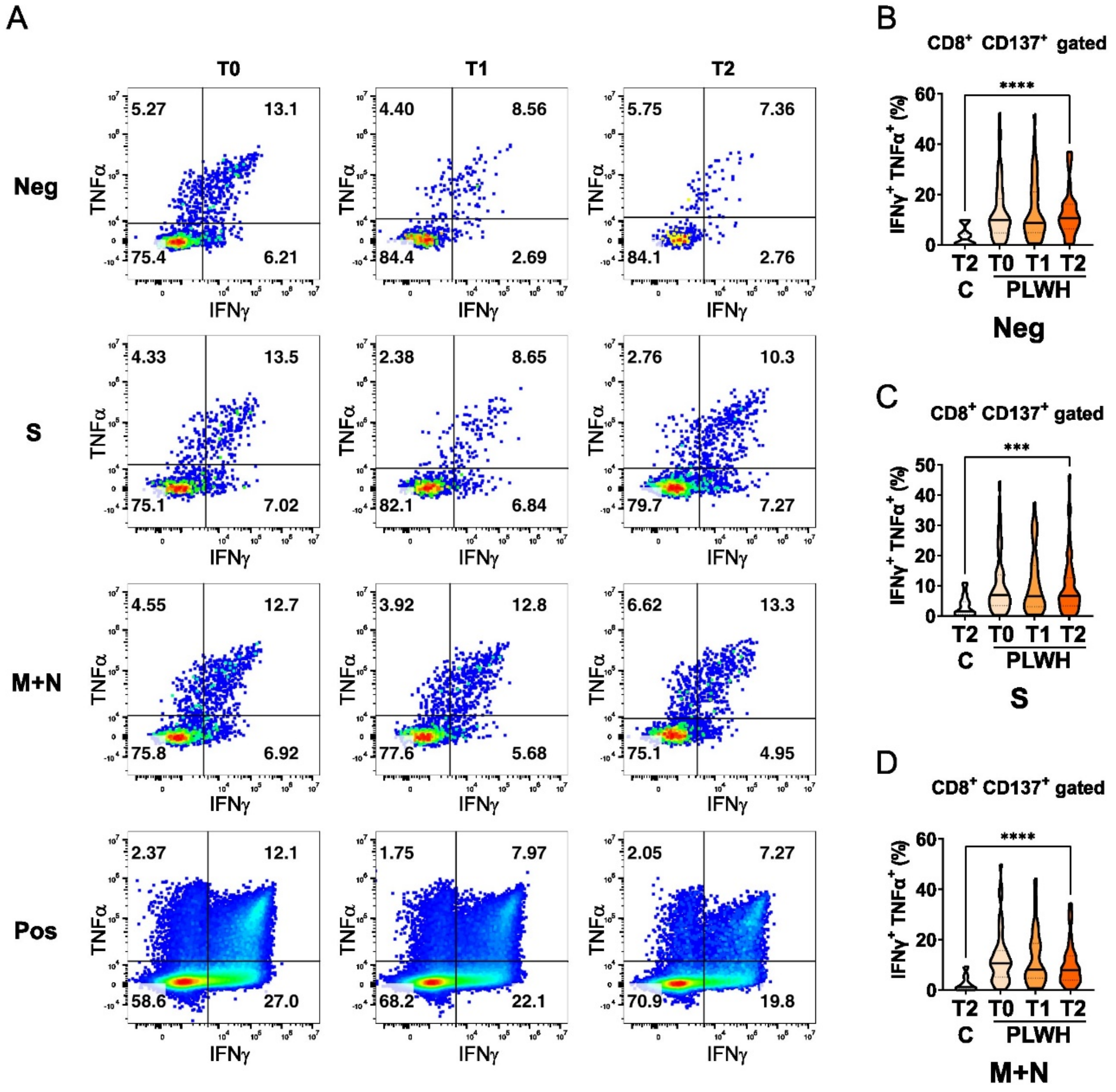

Supplementary Figure 5 Cytokine responses of CD8+ cells from PLWH. **(A)** Representative dot plots of TNFα and IFNγ expression in activated CD8+ CD137+ T cells of a PLWH donor stimulated with SARS-CoV-2 spike (S) glycoprotein peptide pool, or with membrane glycoprotein + nucleocapsid phosphoprotein (M+N) peptide pool, or with CytoStim™ (Pos), or left untreated (Neg) before and after vaccination shots. The violin plots represent **(B)** frequency of activated CD137+ CD8+ T cells that produce the inflammatory cytokines IFNγ and TNFα without any peptide stimulation (negative control) in controls and PLWH, **(C)** frequency of activated CD137+ CD8+ T cells that produce the inflammatory cytokines IFNγ and TNFα upon stimulation with SARS-CoV-2 spike protein-derived peptides (S) in controls and PLWH and **(D)** frequency of activated CD137+ CD8+ T cells that produce the inflammatory cytokines IFNγ and TNFα upon stimulation with peptides derived from SARS-CoV-2 membrane and nucleocapsid proteins that are conserved between different corona viruses (M+N) in controls and PLWH. Statistical significance was calculated by two-tailed Mann-Whitney test: ***$p < 0.001$, ****$p < 0.0001$ (PLWH T0, n= 64-65; PLWH T1, n= 54; PLWH T2, n= 59-61; Control T2, n= 20).

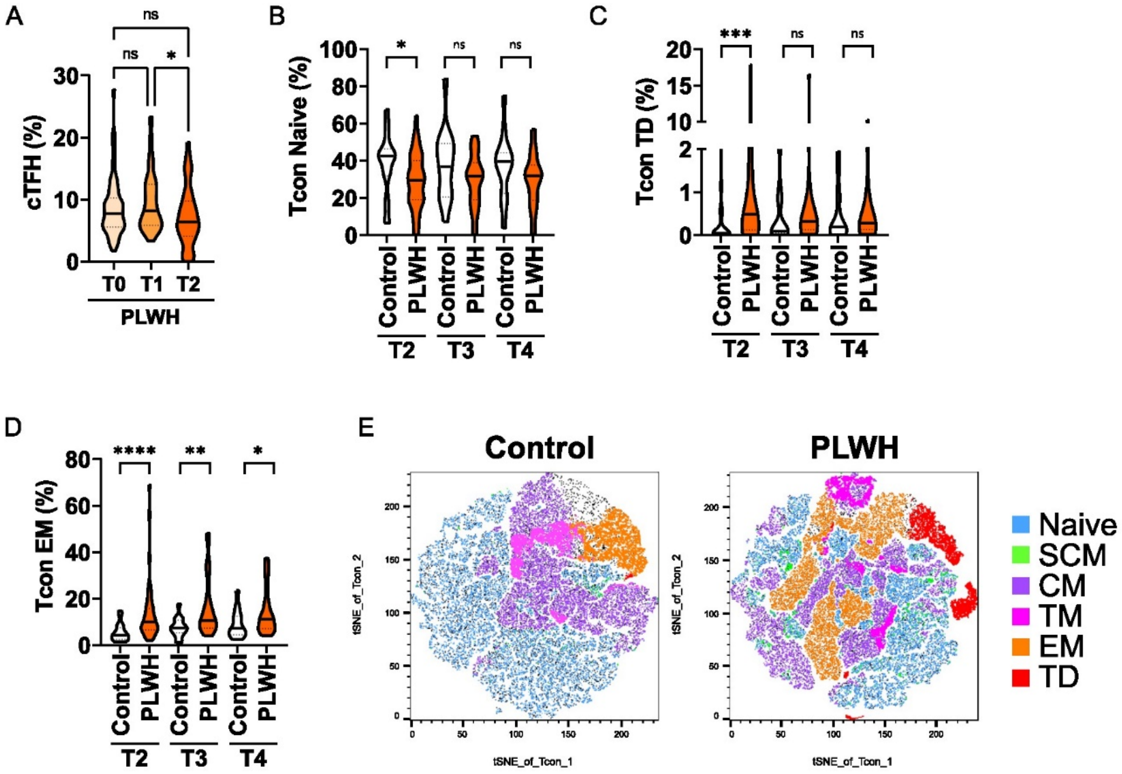

Supplementary Figure 6 Immune phenotyping of CD4+ helper T cells in vaccinated PLWH and HIV-negative controls. **(A)** Frequency of circulating follicular CD4+ T helper cells in PLWH before the first vaccination (T0), before the second vaccination (T1) and 4 to 6 weeks after the second vaccination (T2). Frequencies of Naïve **(B)**, Terminally differentiated (TD) **(C)** and Effector-Memory (EM) **(D)** conventional CD4+ T cells at the indicated time points. **(E)** Representative t-SNE plots of the distribution of naïve, stem cell-like memory (SCM), central memory (CM), transitional memory (TM), effector memory (EM) and terminally differentiated (TD) CD4+ T cells for control and PLWH groups. Statistical significance was calculated by Kruskal-Wallis test and Dunn's test for multiple comparisons (A) or two-tailed Mann-Whitney test (B-D): *p < 0.05, **p < 0.01, ***p < 0.001, ****p < 0.0001, ns = not significant (PLWH T0, n= 65; PLWH T1, n= 54; PLWH T2, n= 64; PLWH T3 & T4, n= 29; Control T2, n= 20, Control T3 & T4, n= 15).

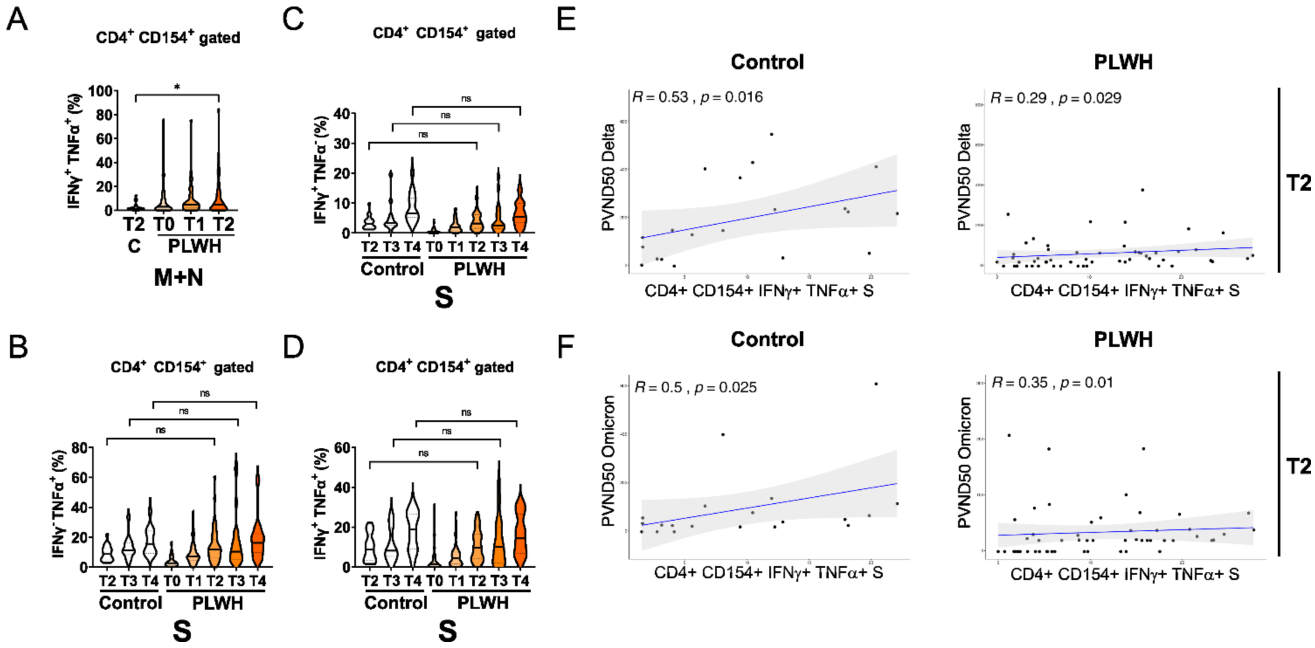

Supplementary Figure 7 Antigen-specific responses of CD4+ helper T cells. **(A)** Frequency of activated, i.e. CD154+, CD4+ T cells that produce the inflammatory cytokines IFNγ and TNFα upon stimulation with peptides derived from SARS-CoV-2 membrane and nucleocapsid proteins that are conserved between different corona viruses (M+N) in controls and PLWH. **(B)** Frequency of activated CD4+ T cells expressing CD154 that produce the inflammatory cytokine TNFα upon stimulation with SARS-CoV-2 spike protein-derived peptides (S) in controls and PLWH at the indicated time points. **(C)** Frequency of activated CD4+ T cells expressing CD154 that produce the inflammatory cytokine IFNγ upon stimulation with SARS-CoV-2 spike protein-derived peptides (S) in controls and PLWH at the indicated time points. **(D)** Frequency of activated CD4+ T cells expressing CD154 that produce the inflammatory cytokines IFNγ and TNFα upon stimulation with SARS-CoV-2 spike protein-derived peptides (S) in controls and PLWH at the indicated time points. Statistical significance was calculated by two-tailed Mann-Whitney test: *$p < 0.05$, ns = not significant. (PLWH T0, n= 64-65; PLWH T1, n= 54; PLWH T2, n= 59-61; PLWH T3 & T4, n= 29; Control T2, n= 20; Control T3 & T4, n= 15). **(E-F)** The spearman's correlation between CD4+ CD154+ IFNγ+ TNFα+ T cells and neutralizing titers against delta (upper) and omicron (lower) variants of SARS-CoV-2 are represented in the scatter plot for healthy participants (left side) and PLWH cohort (right side). R stands for correlation coefficient and p stands for p-value of statistical significance.
